# Gene Expression Switching of Receptor Subunits in Human Brain Development

**DOI:** 10.1371/journal.pcbi.1004559

**Published:** 2015-12-04

**Authors:** Ossnat Bar-Shira, Ronnie Maor, Gal Chechik

**Affiliations:** Gonda Brain Research Center, Bar-Ilan University, Ramat Gan, Israel; Virginia Commonwealth University, UNITED STATES

## Abstract

Synaptic receptors in the human brain consist of multiple protein subunits, many of which have multiple variants, coded by different genes, and are differentially expressed across brain regions and developmental stages. The brain can tune the electrophysiological properties of synapses to regulate plasticity and information processing by switching from one protein variant to another. Such condition-dependent variant switch during development has been demonstrated in several neurotransmitter systems including NMDA and GABA. Here we systematically detect pairs of receptor-subunit variants that switch during the lifetime of the human brain by analyzing postmortem expression data collected in a population of donors at various ages and brain regions measured using microarray and RNA-seq. To further detect variant pairs that co-vary across subjects, we present a method to quantify age-corrected expression correlation in face of strong temporal trends. This is achieved by computing the correlations in the residual expression beyond a cubic-spline model of the population temporal trend, and can be seen as a nonlinear version of partial correlations. Using these methods, we detect multiple new pairs of context dependent variants. For instance, we find a switch from *GLRA2* to *GLRA3* that differs from the known switch in the rat. We also detect an early switch from *HTR1A* to *HTR5A* whose trends are negatively correlated and find that their age-corrected expression is strongly positively correlated. Finally, we observe that *GRIN2B* switch to *GRIN2A* occurs mostly during embryonic development, presumably earlier than observed in rodents. These results provide a systematic map of developmental switching in the neurotransmitter systems of the human brain.

## Author Summary

Synapses change their properties during development affecting information processing and learning. Most synaptic receptors consist of several proteins, each having several variants coded by closely related genes. These protein variants are similar in structure, yet often differ slightly in their biophysical attributes. Switching a synapse from using one variant to another provides the brain with a way to fine-tune electrophysiological properties of synapses and has been described in NMDA and GABA receptors. Here we describe a systematic approach to detect pairs of context-dependent variants at a genome-wide scale based on a set of post-mortem expression measurements taken from brains at multiple ages. We take into account both the profile of expression as it changes along life and also the detrended age-corrected correlation among genes. This method characterizes the landscape of developmental switches in brain transcriptome, putting forward new candidates pairs for deeper analysis. The abundance of switching between context-dependent variants through life suggests that it is a major mechanism by which the brain tunes its plasticity and information processing.

## Introduction

Chemical synapses are complex molecular structures allowing neurons to communicate and process information. In mammals, the molecular composition of most post-synaptic receptors has been characterized, together with their downstream signal transduction pathway [1–3]. Interestingly, many of the key synaptic proteins have multiple variants coded by similar genes, and the actual protein composition of synapses changes across brain regions [4–6], life periods [7,8] and medical conditions [9]. Changing the protein composition of a synapse by switching from one protein variant to another, or changing the fraction of synapses or cells expressing a protein variant, allows the brain to tune various biophysical properties of synapses. These include the temporal profile of synaptic currents, or its plasticity characteristics [10–13]. Presumably, the brain can select which protein variants would be used in a given condition to tune synaptic characteristics to match the condition [14]. We refer here to proteins that have such variants as ***condition***-***dependent variants*** (CDVs).

The idea of protein variants that are utilized differently along development can be well illustrated by the NMDA receptor. This predominant receptor controlling synaptic plasticity has been shown to “switch" its protein composition during development [11,15,16]. NMDA receptors consist of four units. Two of these units are NR1 subunits, expressed in virtually all neurons. The other two non-NR1 subunits, NR2A—D and NR3A-B have a distinct expression pattern through life [11,17,18]. In the prenatal and neonatal rat brain, NMDA receptors predominantly contain NR1/NR2B, but their protein composition later changes such that by adulthood (14–16 weeks) receptors contain mostly NR1/NR2A. The change in subunit composition from NR2B to NR2A affects the kinetics of excitatory post-synaptic currents (EPSCs) [10–12], the binding-site affinity[19] and the sensitivity to pharmacological agents [11,20], together affecting synaptic plasticity and information processing. Age-dependent changes of NMDAR subunit expression were further detected in several brain structures (cortex [12,20,21], cerebellum [5,22], and hippocampus [10]) and species (human [15], mice [23,24]), and were further validated using protein profiling [25]. Similar age-dependent changes have been reported in AMPA [26], Kainate [27], and GABA [28] receptors. Tuning the variant composition of synaptic receptors has been suggested to be a widely used mechanism underlying meta-plasticity [29–31].

Given the evidence for age-dependent changes in protein variant composition, the natural question arises: which synaptic proteins in the human brain switch their variant subunits, at what periods during development, and in which brain regions? Previous studies focused on a handful of molecules in specific brain structures, reporting switches in small groups of proteins and regions. This paper aims to systematically map potential CDVs by detecting pairs of genes whose RNA expression profiles switch through development. Clearly, a switch observed in the expression profile of a gene pair does not necessarily reflect a switch at the protein level, and these candidates have to be further validated using proteomic experiments.

**Fig 1:**
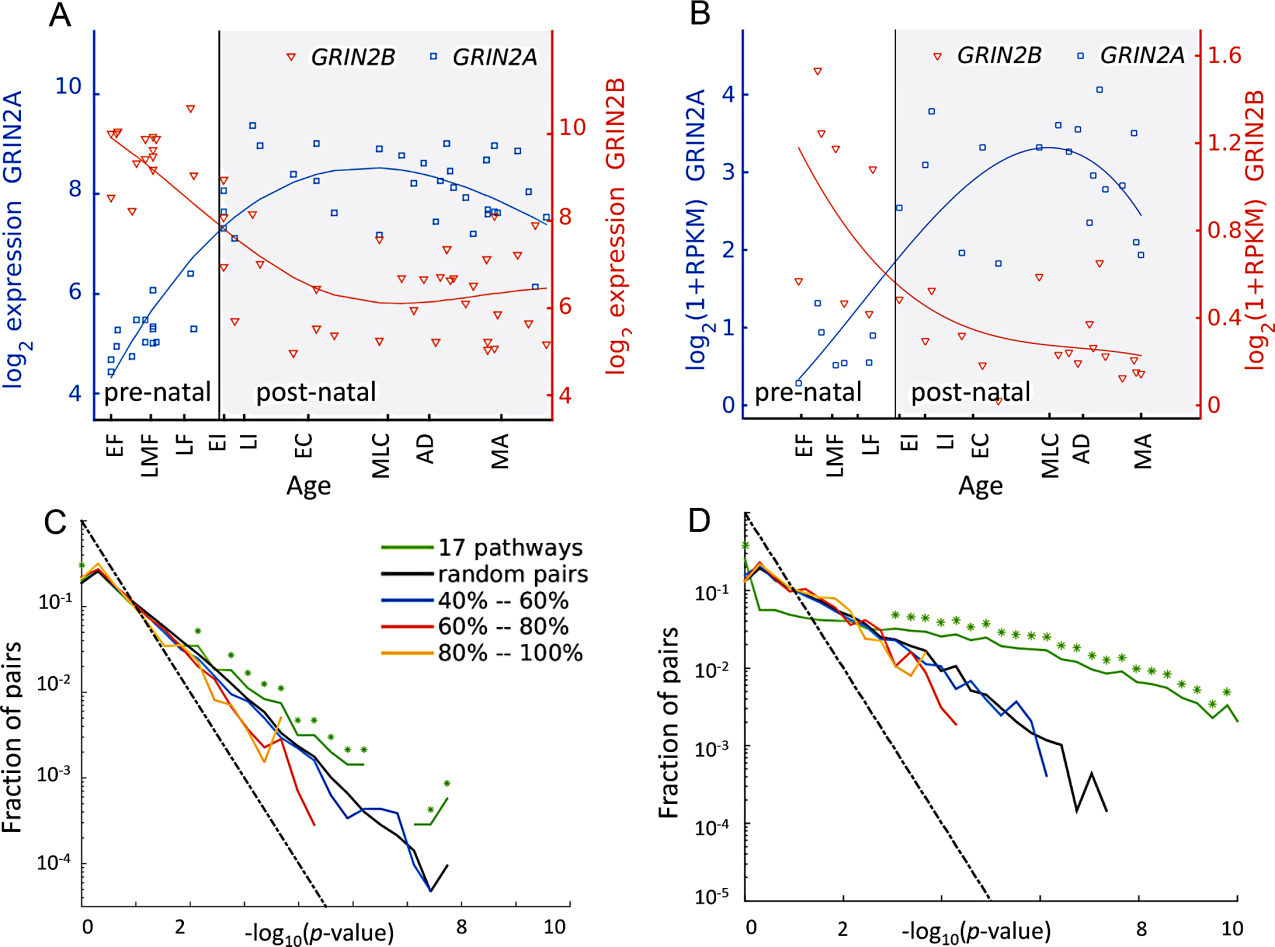
Condition-dependent variants of the NR2 subunits of human NMDA receptors. (A) The expression profiles of GRIN2A and GRIN2B, the two genes encoding NR2A and NR2B in human cerebellum (CBC). The expression level of GRIN2B (NR2B) declines during prenatal and early childhood, while the expression level of GRIN2A (NR2A) rises during that period. (B) Same as A for RNA-seq Brainspan data [34]. (C) Distribution of correlation values across various gene pairs ad regions in the data of [32]. The curve marked ‘pathways’ corresponds to the all pairs from the brain-related keg pathways. Curves marked with percentages correspond to sets of gene pairs whose sequence overlap is in the designated range, based on biomart overlap measure. Asterisks mark bins of the histogram where the observed number pairs in the 17 pathways is significantly larger (permutation test, p <10^–3^) from that obtain at random. Dashed line shows the fraction expected at random. (D) Same as C for RNA-seq Brainspan data [34].

When considering developmental switches, two types of correlations between pairs of genes are of interest. First, as demonstrated in Fig 1, the expression trends of two genes along life may be (anti) correlated. This correlation reflects changes that are driven by age, and are consistent across the population of sampled brains. Importantly, two genes might have opposing or similar ageing trends even if they are not directly co-regulated, simply because they follow opposite trends through life. A second important aspect of switching protein variants is that they may be commonly controlled, either through direct regulatory mechanisms such as transcription regulation, splicing and RNA editing, or through indirect control mechanisms involving brain-wide systems and pathways. Common regulation of a protein pair, either direct or indirect, can be reflected in the two genes fluctuating together from one subject to another. We address these two types of relations when characterizing developmental Switching.

Changes in expression profiles could be interpreted as reflecting several different underlying physiological changes. Since expression is usually measured at the resolution of tissue layer or brain area, expression changes could reflect changes at the level of synapses, cells or tissue. First, ***within-synapse*** changes, where individual synapses change their protein composition such that one protein variant replaces another. Second, ***within-cell*** changes, where individual cells change their synaptic composition such that synapses with some protein variants are replaced by others. Finally, ***within-tissue*** changes, where the proportion or number of cells expressing some protein variants change. In all these cases, the information processing properties of the neural circuit are modified.

Here we take a systematic, genome-wide, approach to search for developmental CDVs by analyzing the temporal gene expression profiles measured in a set of post-mortem human brains of various ages. We study two types of temporal relations among a pair of candidate CDVs: life-long expression trends, and subject-to-subject expression correlation that goes beyond the development trend. The changes captured by this approach may reflect changes in the distribution of synapses within a cell or changes in the distribution of cells in a tissue, and provides candidates for future deeper analysis. We developed a procedure to detect potential CDV pairs, and use it to analyze seventeen brain-related molecular pathways including all the major neuromodulators. The analysis revealed both known and novel CDVs in glycine receptors, where the switching proteins are different from those observed in rodents, and in inhibitory serotonin receptors, providing new evidence for a common regulatory mechanism.

## Results

This paper is organized as follows. We start by illustrating how the known switch of NMDA NR2 variants can be observed in datasets of spatio-temporal gene expression collected in postmortem human brains. Then, we present the two measures of expression correlation, correlation in expression trend along life and age-corrected correlations, as possible evidence for a common regulation mechanism that may underlie the variant switch. Using these two measures, we detect switching protein pairs in seventeen brain-related molecular pathways. Finally, we discuss in detail new potential CDVs and their possible functional implications.

The results are based on analysis of three datasets. The first dataset contains the transcriptome of 17,565 mainly protein-coding genes collected by Kang *et al*. [32] from 11 cortical and 5 sub-cortical brain regions of 57 human donors. The second dataset contains transcriptome profiles collected using microarrays from the prefrontal cortex of 269 human donors, collected by Colantuoni *et al*. [33]. The third contains RNA-seq data for 16 brain regions collected by the Brainspan consortium [34].

### NMDA NR2 subunits switch in early human brain development

We start by demonstrating that a switch between two NMDA receptor subunits, NR2B and NR2A is reproduced in the transcriptome of postmortem human brains measured with microarrays and RNAseq. In rats, NR2A and NR2B exhibit prominent switching in the cerebellum during development, both at the mRNA and protein levels [5,22,25,35]. Here we traced the expression levels of the human genes *GRIN2A* and *GRIN2B*, encoding for the protein variants NR2A and NR2B, as measured in 57 human brains (see methods). Fig 1A and 1B shows a clear “switch” from *GRIN2B* to *GRIN2A* in the cerebellum, consistent with the switch reported in rodents. The switch is occurs around birth and does not occur in other brain regions, where *GRIN2B* maintains high expression level while *GRIN2A* rises.

### Detecting condition-dependent variants (CDVs)

We now proceed to map systematically the synaptic proteins which exhibit a developmental switch from one variant to another. Clearly, considering all protein pairs is wasteful and would yield many false positives. We therefore limit the set of candidate protein pairs using prior knowledge about their functional role and sequence similarity. Specifically, we used the KEGG pathways repository [2] to focus on proteins that participate in synaptic pathways, including signal transduction pathways of all neurotransmitters (a total of 17 KEGG pathways). We then used KEGG to define subgroups of functionally-related proteins which are likely to contain CDVs (see Methods). Then, we calculated the similarity of the two amino-acid sequences of each candidate pair, and kept pairs with sequence similarity above 30% (see Methods). Low sequence similarity is usually associated with different functionality and dissimilar protein sequences can be excluded from consideration [40].

For all protein pairs remaining as candidate CDVs, we compute two measures of correlation for every brain region: the correlation of the two expression profiles along life and the age-corrected correlation capturing subject-to-subject fluctuations. We now describe separately the top-ranked protein pairs for each of these two measures, and later discuss in detail two specific pairs which have significant correlation in both.

### Anti-correlated CDVs in brain-related pathways

We considered gene pairs coding for proteins in seventeen brain-related pathways. For each pair of proteins, we calculated the correlation of their expression profiles as measured in from three datasets: Kang *et al*. (2011) for 16 brain regions using mRNA levels measured using microarrays across 57 subjects; Colantuoni *et al*.(2011) for one brain region with 269 subjects; and RNA-seq data for 16 brain regions provided by Brainspan (see Methods). Overall, we examined 843 unique pairs in 17 brain regions, 8.3% of which have a statistically significant anti-correlation (FDR-corrected Pearson correlation *q*-value<0.01).

To test if this abundance of correlated pairs is significant, we repeated the calculation using other groups of gene pairs. Specifically, we computed the correlation among all paralog gene while grouping pairs by their sequence similarity, and also using random pairing of these paralog genes pairs (see Methods). Fig 1C and 1D shows that the expression of paralog genes, regardless of their sequence similarity, has a similar distribution of correlations as the expression of random pairs (from the same set of paralog genes). However, significantly many more pairs selected from the KEGG pathways to be functionally related exhibit strong correlations. This result suggests that many functionally-related pairs of genes in brain-related molecular pathways exhibit a development switch.

**Fig 2:**
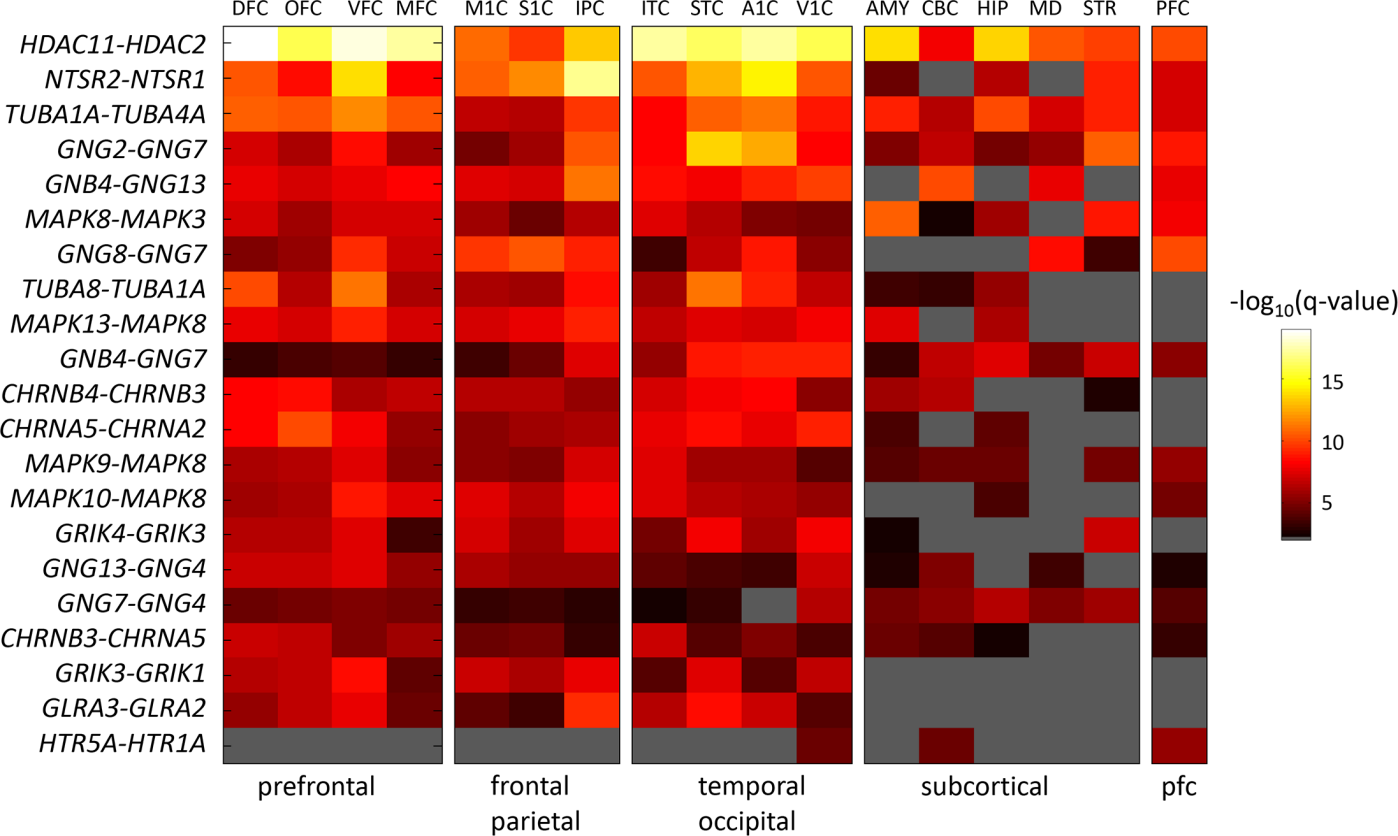
Dissimilarity significance of the top 20 genes in 17 brain regions measured by Kang *et al*. [32] and in the prefrontal cortex measured by Colantouni *et al*. [33]. Colors correspond to negative *log*_*10*_(*p*-values) of Pearson correlation. Gray pixels denote regions in which the range of expression levels (maximum-minimum) was below 1.5 for at least one gene, or an insignificant *q-value*. The bottom row shows dissimilarity for an interesting pair of serotonin receptors. Brain regions are grouped and sorted as follows: prefrontal: DFC, OFC, VFC, MFC; frontal-parietal: M1C, S1C, IPC; temporal-occipital: ITC, STC, A1C, V1C; subcortical: AMY, CBC, HIP, MD, STR; prefrontal data from [33]: PFC. Region codes are listed in the Methods section. The genes coding for histone deacetylase, *HDAC2* and *HDAC11* exhibit significant dissimilarity levels in all brain areas. The genes coding for Neurotensin receptor *NTSR1* and *NTSR2* exhibit significant dissimilarity mainly in cortical regions and the striatum.

To further look into the specific switches found, Fig 2 gives the negative log_10_ of the FDR *q*-values for the top 20 genes in the 17 brain regions examined, ranked by the average score across 17 regions, based on microarray measurements. A similar figure based on RNA-seq data is given in S2 Fig. The two data sources largely agree (S3 Fig). The full list of all significant CDV pairs is given in a supplemental webpage at http://chechiklab.biu.ac.il/~ossnat/brain-paralogs/.

Many of the top-ranked human CDVs pairs are consistent with experiments in rodents or previous literature. For instance, our analysis reveals a significant switch of the human genes *GRIN2B* and *GRIN2C* in the cerebellum starting at the late fetal stage LF7 (FDR-corrected Pearson correlation *q*-value <10^−10^, see S1A and S1C Fig). This is consistent with previous reports of a switch of *GRIN2B* and *GRIN2C* in rodent cerebellum during early development [5,16]. In rodents, the switch occurs in cerebellar granule cells, after their migration from the external germinal layer to the inner granular layer [38].

As another example, in rats, *in-situ hybridization* studies of the cerebellar granule cells have detected a pronounced increase in the expression and contribution to postsynaptic receptors of the GABA receptor α6 subunit in the first two postnatal weeks (between P6 and P14) [4,39,40]. This increase was limited to internal granule layer [4,39,40]. The expression of the α3 subunit has declined in this time period [4]. Here we found a rise in the RNA levels of *GABRA6*, the gene encoding for the α-6 subunit of the GABA receptor, with a parallel decrease in RNA levels of *GABRA3*, the gene encoding the α-3 subunit (S1B Fig, *q*-value <10^−12^). Other subunits may be changing as well.

### Age-corrected subject-to-subject expression correlation

The above results point to numerous protein pairs whose expression profile in the population shows anti-correlated trends, as in a developmental switch. We now turn to study how the expression of these genes fluctuates around these trends. When two genes fluctuate together, as illustrated in S5 Fig, it means that their expression changes in a coordinated way from one subject to another. Such coordination could be due to a direct regulatory mechanisms such as transcription regulation, splicing and RNA editing, or through indirect control mechanisms involving other systems and genes and operating at a slow timescale. Importantly, the two types of correlations we analyze, namely correlation of expression trends and correlation of fluctuations around the trend, are independent in the sense that the presence of trend correlation does not necessarily lead to subject-to-subject correlations and vice versa. The two types of correlations could reflect different underlying mechanisms.

Computing the residual correlations of non-stationary signals has been previously studied in various domains [41]. Commonly, the trend is modelled in a parametric or non-parametric way, which allows subtracting the underlying population trend. Often, trends are assumed to be linear, and the method of partial correlations is used to compute the trend-corrected fluctuations (see Methods for details). The expression trends in our data are far from linear, and we therefore take a non-linear detrending approach.

**Fig 3:**
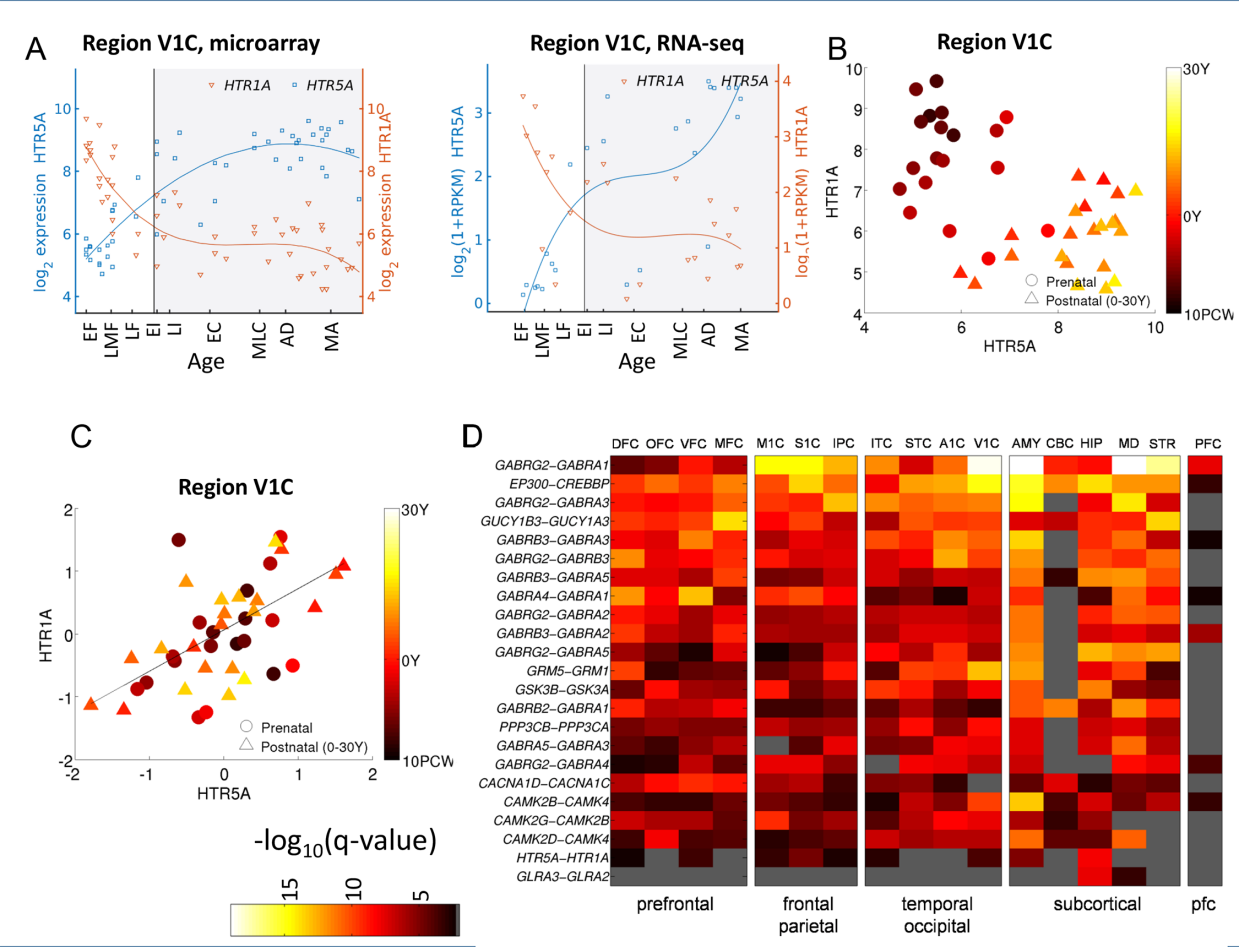
Age-corrected correlation. **(A)** An example of fitting a cubic spline[80] to the expression of *HTR1A* and *HTR5A* in the primary visual cortex (V1C). Circles denote measured data points and solid lines denote a cubic spline fit. **(B)** Expression levels of *HTR1A* and *HTR5A*. Circles denote the expression of prenatal subjects, triangles of postnatal. The color scale denotes the age of each donor, with older subjects in light yellow and young subjects in dark. The expression is anti-correlated (ρ = -0.58). **(C)** After removing the population effect of age, the expression of *HTR1A* and *HTR5A* is positively correlated across subjects (ρ = 0.53, *q*-value < 10^−4^, Pearson). **(D)** The magnitude of age-corrected correlation (residuals) of 20 CDV pairs, ranked by the mean correlation over regions.

To estimate age-corrected correlations in development expression data while correcting for the possible effect of age, we estimated the *underlying population trend* by fitting a model to the temporal profile of each gene, based on the expression in the population. Specifically, after testing several models, we used here a cubic spline model (See Methods). Fig 3 illustrates how correcting for age reveals significant correlations among gene pairs. First, Fig 3A depicts an example of a spline fit to *HTR1A* and *HTR5A*. Fig 3B and 3C describe age-corrected correlation between the expression levels of the two genes before and after correcting for trend due to age. While the correlation before correcting for the trend is negative (*ρ = -0*.*58*, FDR corrected Pearson correlation *q-v*alue = *2*.*5·10* ^*−5*^), the age-corrected correlation is strongly positive (*ρ = 0*.*53*, *q*-value = *2*.*2·10* ^*−4*^). The positive age-corrected correlation is therefore masked by the negative correlation induced by the trend. One model that is consistent with this finding is that the two genes share a common regulation mechanism that affects the mRNA levels of both genes in a similar way.

We repeated computing age-corrected correlations for all candidate gene pairs. Fig 3D provides a heat map with the magnitude of age-corrected correlation, measured using log_10_(*q*-value), for the top 20 correlated pairs and also for additional pairs that are discussed in detail below.

Estimating age-corrected correlations can be sensitive to various biases. First, variability in RNA quality or density across subjects could yield spurious correlations that are not limited to the set of genes studied. To estimate the magnitude of this effect, we computed age-corrected correlations in a set of house-keeping genes (HKG) [42], and found that strongly correlated HKG pairs are significantly less common than observed in pathway genes (S6 Fig). This suggests that the correlations we observe are significantly more abundant in the pathway genes we studied than in random gene pairs. Correlations could also stem from fluctuations across subjects in the fraction of neurons sampled. Our estimates show that such variability is not likely to explain the strong correlation effects that we observe (Supplemental results).

Together, the two correlation measures discussed above, anti-correlation along life and age-corrected correlation, yield a large number of gene pairs with trends that are anti-correlated, and a significant age-corrected correlation (see full list in supplemental webpage). We now discuss in detail two such examples: glycine receptors and serotonin receptors.

### Glycine receptor switch in humans differs from rodent switch

**Fig 4:**
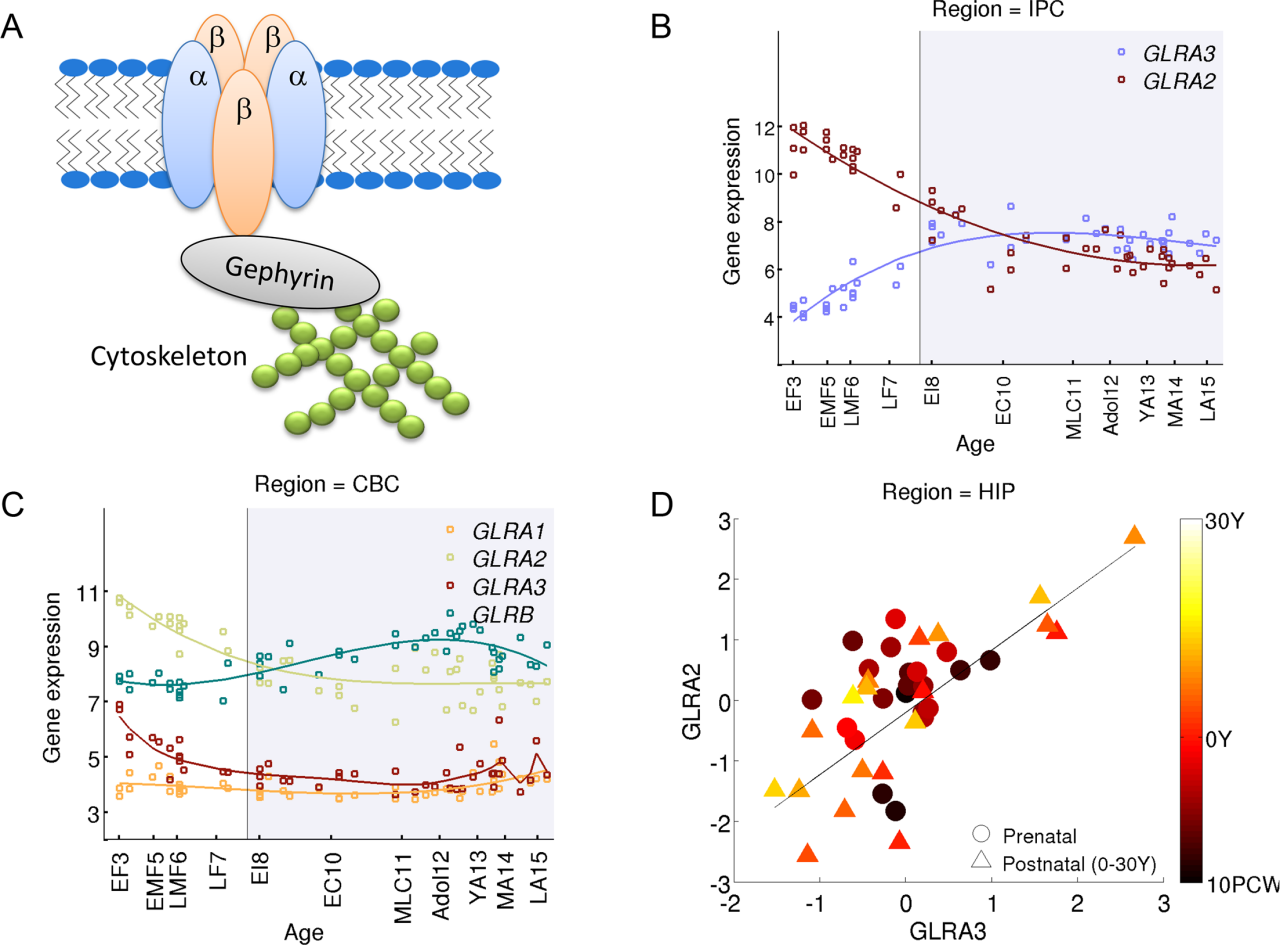
A novel CDV pair in the glycine receptor subunits GLRA2 and GLRA3. **(A)** A diagram of glycine receptor components **(B)** The gene expression profiles of glycine receptor subunits as measured by Kang et al. (2011) in the human posterior inferior parietal cortex (IPC). **(C)** The gene expression profiles of glycine receptor subunits as measured by Kang et al. (2011) in the human cerebellar cortex. **(D)** Subject to subject age corrected correlation within two age groups, prenatal (10PCW-birth), and postnatal (0-30Y).

Glycine is a major inhibitory neurotransmitter in the central nervous system, operating by causing an influx of chloride which hyperpolarizes a cell when glycine receptors are activated. Disruption in glycine receptor expression or ion channel function can result in hyperekplexia, a rare neurological disorder. Glycine receptors (GlyRs) could include two subunits: α, which has four variants α1, α2, α3, α4, and β, which has a single variant, and they appear as pentameric α homomers or as αβ heteromers[43]. The β-subunit binds gephryn [44,45], a protein that anchors the glycine receptor to the cytoskeleton (Fig 4A), and allows clustering of heteromeric glycine receptors at the synapse [46–48]. Heteromeric GlyR likely accounts for most of the GlyRs in the adult CNS [43]. Homomeric α GlyR still form functional GlyRs [49], and is found in embryonic neurons [50], but since they do not bind gephryn, homomeric α1-α3 GlyRs are most likely to be extrasynaptic [43]. In humans, the gene encoding for α4 is a pseudogene and is not expressed [51], but all other subunits were found to be expressed in several brain regions including the cerebral cortex [52], the striatum [53] and the amygdala [54].

In the rat, GlyRs have been shown to switch from homomeric α2 receptors to heteromeric α1β receptors by postnatal day 20 [13]. As a result most glycinergic neurotransmission in the adult rat brain is mediated by α1β receptors [43], composed of three α1 and two β subunits [47,55].

Here we find that human GlyRs follow a different pattern, switching from *GLRA2* to *GLRA3*. This switch is significant across the cortex (*q*-value <0.01, Figs 2 and 4B, Supplemental webpage), but not in the cerebellum, where the expression of both subunits decreases with age (Fig 4C), or the thalamus. Since synapses containing homomeric α3 GlyRs are weakly expressed in the mature vertebrate brain [43], it is possible that the switch we detected in the expression levels of *GLRA2* and *GLRA3* reflects a shift from homomeric α2 GlyRs to heteromeric α3β GlyRs, similar to the switch reported from α2 to α1β in rats. This hypothesis is supported by the expression profile of *GLRB*, which is highly correlated (*p*-value <0.01) with the profile of *GLRA3* in all brain structures except the cerebellum and the striatum (for example, the correlation coefficients in the IPC, ρ = 0.70, *p*-value < 10^−8^ and in the amygdala ρ = 0.81, *p*-value <10^−11^, Pearson).

Furthermore, *GLRA2* and *GLRA3* have strong positively age-corrected correlation in the hippocampus (*q*-value < 10^−7^, Fig 4D), and other regions (mediodorsal nucleus of the thalamus, *q*-value = 9.05·10^−4^; amygdala, *q*-value = 0.0062). This suggests that *GLRA2* and *GLRA3* might be commonly regulated (directly or indirectly) in the postnatal human brain.

### Serotonin switch in HTR1A and HTR5A

A second example of a novel switch is the pair of serotonin-receptor genes *HTR1A* and *HTR5A*. Serotonin is a major neuromodulator involved in regulation of mood [56–58], aggressive behavior [59], and sleep–wake cycle [60,61]. Importantly, serotonin is involved in major mood pathologies like depression and aggressive behavior, and selective serotonin reuptake inhibitors (SSRIs) are today the most widely used anti-depressants. There are seven families of serotonin receptors, each containing multiple subunits. We focus here on two specific proteins 5-HT_1A_ and 5-HT_5A_ which are both compounds of G_αi_-coupled inhibitory receptors 5-HT_1_ and 5-HT_5_.

The two receptors 5-HT_1_ and 5-HT_5_ share many properties [62]. 5-HT_1_ acts as an autoreceptor on serotonergic neurons [63,64], and mediates hyperpolarization of 5-HT on prefrontal neurons [65]. 5-HT_5_ receptors activate the same signal transduction pathways as 5-HT_1_ through G-α_i_ coupled protein [62]. 5-HT_5_ receptors were found to act as autoreceptors [66] and induce inhibitory influence on neuronal excitability [67]. The areal expression pattern of both 5-HT_1A_ and 5-HT_5A_ is similar, as they are both expressed in the amygdala, the cerebellum, the hippocampus, and in cortical layers III and V. Both receptors are highly expressed in pyramidal neurons in the cerebral cortex and in the hippocampus [68,69].

**Fig 5:**
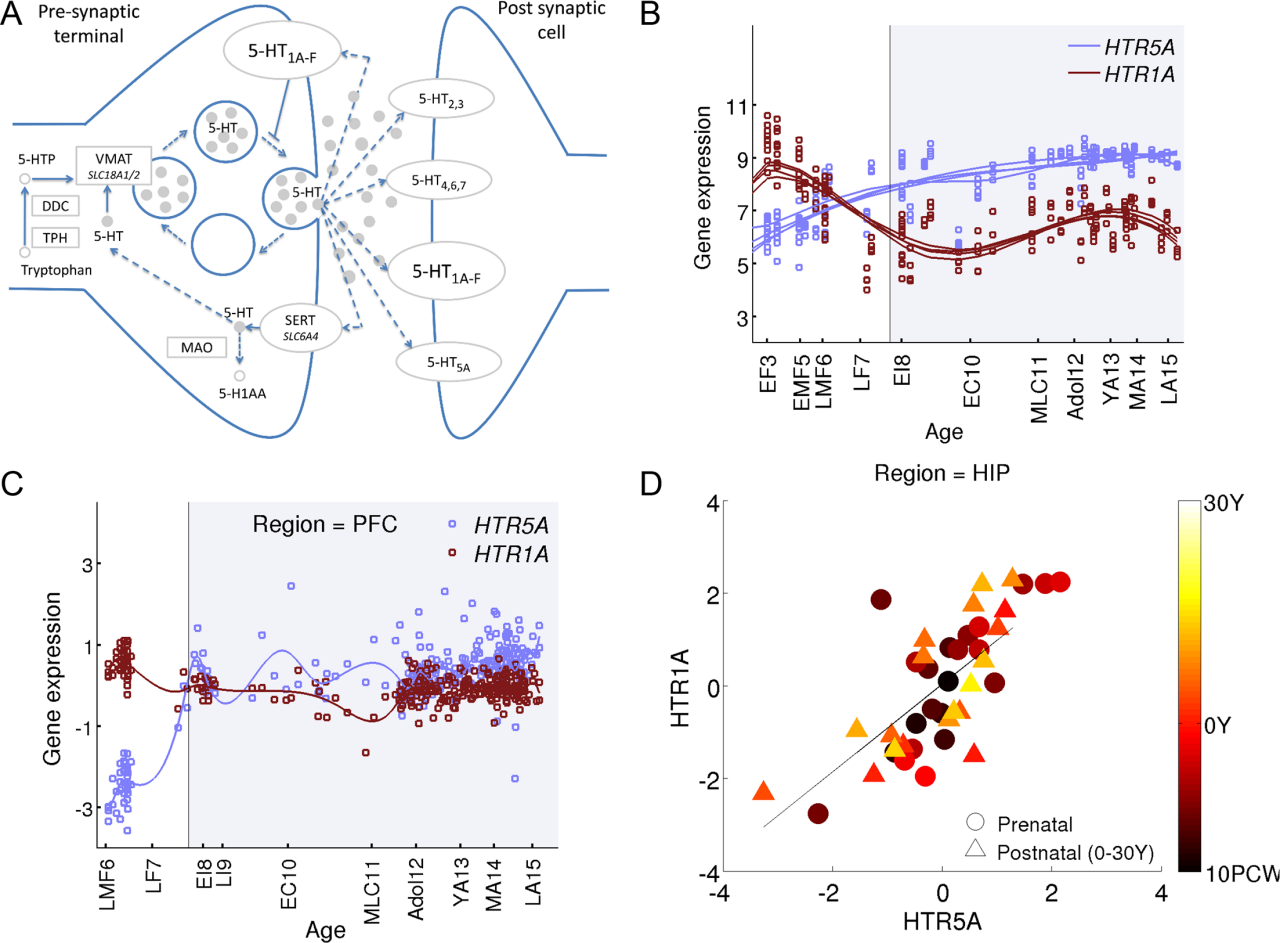
A novel CDV pair of serotonin-receptor genes *HTR1A* and *HTR5A*. **(A)** A diagram of the serotonergic synapse. **(B)** The gene expression profiles of *HTR1A* and *HTR5A* serotonin receptors as measured by [32] in four regions of the human prefrontal cortex: dorsolateral, medial, orbital and ventrolateral prefrontal cortex. **(C)** The gene expression profiles of *HTR1A* and *HTR5A* serotonin receptors as measured by [33] in the prefrontal cortex. **(D)** Subject to subject correlation, after removing the population effect of age, between the expression levels of *HTR1A* and *HTR5A* within two age groups, prenatal (10PCW-birth), and postnatal (0-30Y) in the hippocampus.

We find that in the prefrontal cortex the expression of *HTR1A* significantly decreases during early embryonic development while the expression of *HTR5A* increases (Fig 5B). This is also observed in a second human postmortem dataset collected in the prefrontal cortex [33] (Fig 5C). This switch is significant in the primary visual cortex (FDR *q*-value<10^-4^), and in the prefrontal cortex (*q*-value<10^-5^). These findings complement a recent study by Lambe and colleagues, who analyzed serotonin expression from 59 human subjects aged 6 weeks to 50 years [70]. They reported a developmental increase of *HTR5A* expression in the human prefrontal cortex, paralleled by a stable expression of *HTR1A* during post-natal development [70]. The analysis here shows that these findings continue a trend that started during embryonic development.

When correcting for the population trend across life, *HTR1A* and *HTR5A* are significantly positively correlated in seven out of sixteen brain regions, including the hippocampus (*ρ* = 0.725, *q*-value <10^-7^, Fig 5D), the primary visual cortex (*ρ = 0*.*53*, *q*-value *<*10^-3^, Fig 3C) and other cortical areas (see Supplemental webpage). At the same time, they are slightly negatively correlated in the cerebellum (ρ = -0.3, *q*-value = 0.05), striatum (ρ = -0.048), and prefrontal cortex (ρ = -0.1). This suggests that the two genes might be commonly regulated, and that this regulation may depend on brain region.

### NMDA GRIN2A/GRIN2B switch occurs early in human

As discussed above, the developmental switch of the NR2 subunit of the NMDA receptors from NR2B to NR2A has gained significant interest, since it affects the functional properties of the NMDA synapse, possibly reducing its plasticity. In rodents, both NR2B and NR2A represent an important fraction of juvenile and adult NMDARs [14]. We therefore turned to look into the details of the NR2A/2B switch in three brain regions: Cortex, Hippocampus and Cerebellum.

**Fig 6:**
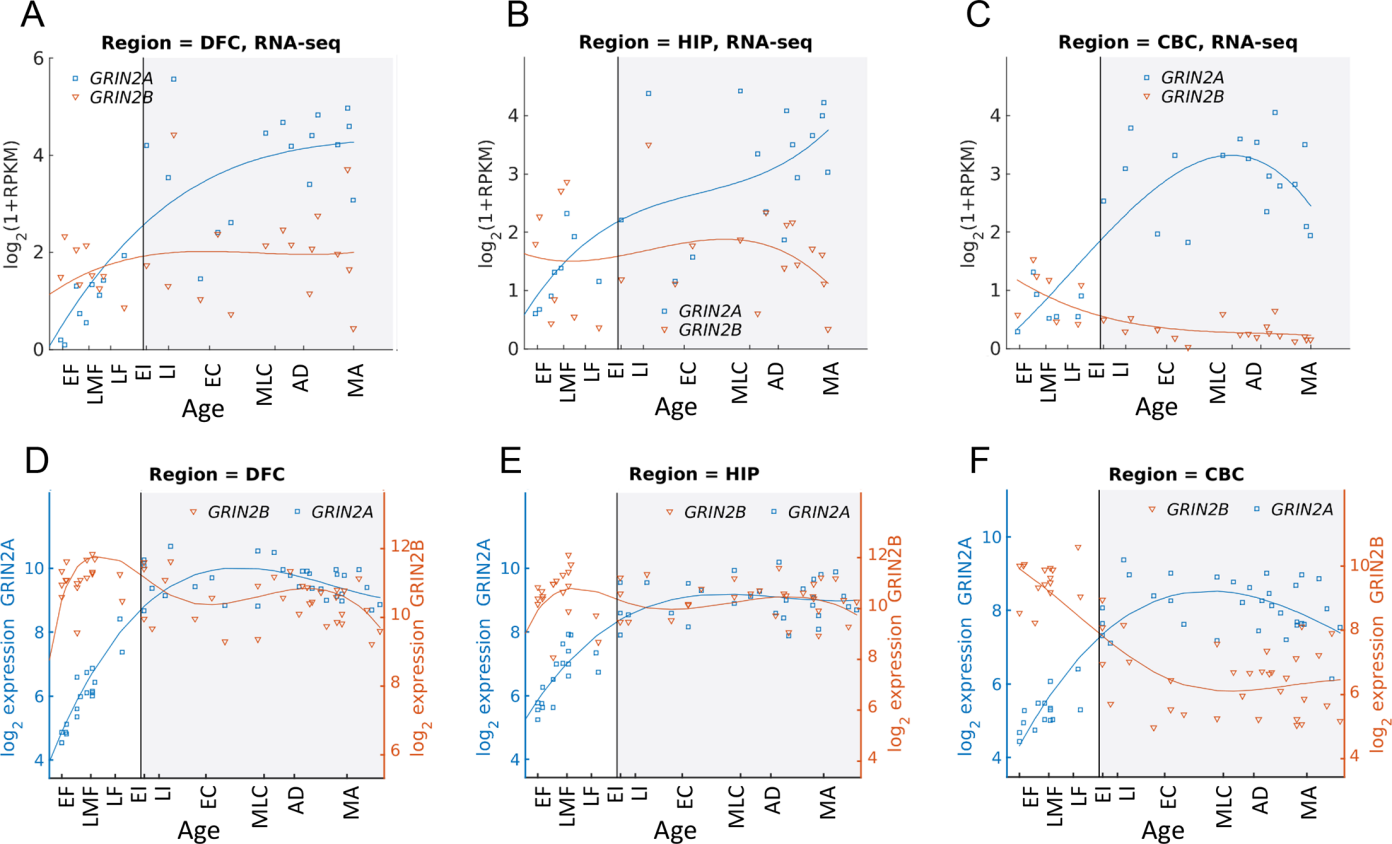
Timing and spatial differences of the NR2A/2B developmental switch. In human cortex (RNA-seq:A, microarrays: D) and Hippocampus (RNA-seq:B; microarrays:E) childhood expression level of NR2B exhibit little significant change, and NR2A may be rising slowly. In the cerebellum (RNA-seq: C; microarrays: F), a more significant switch is observed. In all cases, the largest changes in expression levels occur before birth, and much smaller changes are observed postnatally.

Fig 6 shows the expression profiles of *GRIN2A* and *GRIN2B* as measured using RNA-seq (top row) and microarrays (bottom row, [32]) for three brain regions: The dorsolateral prefrontal cortex (DFC), hippocampus (HIP) and the cerebellum (CBC). In the cortex (Fig 6A and 6D) and hippocampus (Fig 6B and 6E), *GRIN2B* levels appear to remain at the same level throughout life, while GRIN2A levels rise during embryonic development and remain steady (microarrays) or rise slightly (RNA-seq) after birth. In the cerebellum (Fig 6C and 6F), *GRIN2B* levels slowly decline throughout life, and GRIN2A levels rise abruptly around birth, such that postnatal levels are higher than prenatal ones. It should be noted that the RNA transcript counts may not reflect protein levels directly, since different genes may have different degradation and translation rates, such that the number of protein molecules per RNA may vary across genes.

Interestingly, it appears that in humans, the time of the developmental switch is early, and does not extend into childhood or adolescence as observed in rodents [14,24] where changes of expression patterns occur within the first two postnatal weeks [22]. This could suggest that in humans, the switch from NR2B to NR2A is not related to the decrease in behavioral plasticity observed during late childhood and puberty. This topic awaits further experiments and analysis which is beyond the scope of the current paper.

## Discussion

Developmental switching of protein variants that function as subunits of synaptic receptors is a mechanism that allows the brain to tune functional properties of synapses [71,72] and underlies meta-plasticity [29–31].

Here we described a systematic way to find context dependent variants (*CDV*s) that switch during development. The procedure we propose detects pairs of substitutable proteins, based on their structure and function similarity and the dissimilarity of their abundance profile. We used this method to detect candidate pairs of *CDV*s in elements of seventeen brain-related pathways. We investigated more deeply two pairs of candidate *CDV*s. First, we found that human glycine receptors switch from α2 to α3 subunits. This switch differs from previous results in rodents, and raises the hypothesis that glycine receptors in the adult human brain contain α_3_β heteromers. Second we find a switch of serotonin receptors 5-HT_1A_ to 5-HT_5A_ in the cerebellum and the hippocampus. These two serotonin receptor proteins co-vary across subjects exhibiting high age-corrected correlations and suggesting that they may be directly or indirectly regulated.

Our results are based on a coarse measure of average expression levels in a region. As such, they are limited in several ways. First, mRNA levels may not reflect protein levels in these brain areas, hence these results should be viewed as providing candidates for future proteomic measurements. Importantly however, since the trend correlations that we observe are based on changes of expression level within a gene through life, the results are invariant to any linear transformation of the expression measurements. Second, multiple variants may be switching concurrently, and the analysis can be extended beyond pairs of genes. Third, a switch in aggregated mRNA level measured as an average in a brain tissue could result from changes at various levels. It could reflect a process by which cells start expressing a different mix of subunits in their receptors, or express a different mix of receptors. It is also possible that the *mixture of cells* in the tissue changes, such that cells expressing one receptor become more abundant. In the context of the above findings, such changes in cell composition are particularly likely in early developmental stages in the cerebellum, where neural migration and wiring matures later than in other areas. Regardless of the level where the developmental switch takes place, it could affect the information processing and plasticity properties of the network.

The analysis in this paper focused on switches at the gene level. Recent RNA sequencing measurements now allow extending it to exon level, which could detect context-dependent splice variants. Indeed, there is evidence that switching between splicing alternatives is a key event in neuron differentiation [73], and a systematic study of developmental changes in splicing in human and mammalian brain would be of great interest.

The vast majority of switches that our analysis detected occurred around late embryonic development, and many have stretched into infancy and childhood. This is consistent with the massive changes in expression levels that many genes exhibit around birth [32,33]. This can have important implications regarding the stability of early learned experience, since it suggests that many synaptic connections get replaced around birth, possibly affecting the capacity of the network to retain early experiences. Quantifying the shapes of the transition curves and the subject-to-subject interactions among subunits of the receptors can shed further light on how subunits are utilized at various developmental stages. This can benefit from using parametric models of curve shapes to capture timing information [74].

The two pairs of switching CDVs we discussed, serotonin and glycine receptors, are characterized by a strong anti-correlated population trend, together with age-corrected correlation which was positive in many regions. This demonstrates that the regulation of these pairs involves various control mechanisms operating on multiple scales. The precise nature of the regulation mechanisms operating on these pairs awaits further study.

## Methods

### Postmortem human transcriptome data

We analyzed three gene expression datasets. The first dataset was collected by Kang et al. using microarrays from the brains of 57 human donors [32]. The dataset contained transcriptome of 17,565 mainly protein-coding genes collected from 11 cortical and 5 sub-cortical brain regions. We included all subjects older than 10 post conceptual week (PCW), leaving a total of 53 subjects aged 10PCW to 82 years. The data were originally quantile-normalized and log_2_-transformed. When presenting results, we followed the classification of subjects into 13 age groups made in [32] and specified in Table 1. Classification into age groups was not used in the analysis.

**Table 1:** Donors age groups following [32].

Age group	Description	Age
EF3	Early fetal	10 PCW ≤ Age < 13 PCW
EMF4	Early-mid fetal	13 PCW ≤ Age < 16 PCW
EMF5	Early-mid fetal	16 PCW ≤ Age < 19 PCW
LMF6	Late-mid fetal	19 PCW ≤ Age < 24 PCW
LF7	Late fetal	24 PCW ≤ Age <38 PCW
EI8	Neonatal and early infancy	0 M (birth) ≤ Age <6M
LI9	Late infancy	6 M ≤ Age < 12 M
EC10	Early childhood	1 Y ≤ Age <6 Y
MLC11	Middle and late childhood	6 Y ≤ Age < 12 Y
Adol12	Adolescence	12 Y ≤ Age < 20 Y
YA13	Young adulthood	20 Y ≤ Age < 40 Y
MA14	Middle adulthood	40 Y ≤ Age < 60 Y
LA15	Late adulthood	60 Y ≤ Age

The second dataset, collected by Colantuoni *et al*. [33], contains mRNA microarray measurements of 30,176 genes in the prefrontal cortex of 269 human subjects. Donor ages range from 18PCW to 78 years. When computing correlation *p*-values this data was sub-sampled uniformly to bring all results to a common scale.

The third dataset, collected by the Brainspan consortium and described in [34], contains RNA sequencing measurements collected from the same set of brain tissues as [32]. We downloaded the version available on the Brainspan website on November 2014 (Gencode v10 summarized to genes).

### Pathways

In this work we considered all pathways in the KEGG pathway collection [1] that are classified under *Nervous system*, *Substance dependence*, and *Neurodegenerative diseases*. We also examined the pathway *Neuroactive ligand-receptor interaction*, which contains G-protein-coupled receptors and ion channels. Overall we analyzed seventeen pathways listed in Table 2, all retrieved from the KEGG pathway repository (www.genome.jp/kegg/pathway.html) on September 2012.

**Table 2:** List of KEGG brain-related pathways used to search for potential CDVs.

Pathway	KEGG accession ID	KEGG category
1. Long-term potentiation (LTP)	04020	Nervous system
2. Glutamatergic synapse	04724	
3. Cholinergic synapse	04725	
4. Serotonergic synapse	04726	
5. GABAergic synapse	04727	
6. Dopaminergic synapse	04728	
7. Long-term depression	04730	
8. Alzheimer's disease	05010	Neurodegenerative diseases
9. Parkinson's disease	05012	
10. Amyotrophic lateral sclerosis (ALS)	05014	
11. Huntington's disease	05016	
12. Cocaine addiction	05030	Substance dependence
13. Amphetamine addiction	05031	
14. Morphine addiction	05032	
15. Nicotine addiction	05033	
16. Alcoholism	05034	
17. Neuroactive ligand-receptor interaction	04080	Signaling molecules and interaction

### Extracting groups of functionally related proteins

To reduce the fraction of false positives, we limited candidate protein pairs to proteins that reside within the same functional element in KEGG pathway repository [2]. These KEGG elements group together proteins with common functionally and interaction partners. Often, but not necessarily, these proteins belong to the same family. We also tested filtering candidate proteins based on protein families, but found that KEGG elements were usually more functionally-coherent than protein families, and at the same time less specific than protein sub-families. In many cases, members of the same family that are functionally distinct are split into separate pathway elements. For example, the family of *Glutamate-gated ion channels* (UniProtKB) includes all the subunits of NMDA, AMPA and Kainate receptors. In KEGG’s *Glutamenergic synapse* pathway, this family is split into three elements: KAR, AMPAR and NMDAR—one per receptor type.

### Excluding pairs with low sequence similarity

In some cases, proteins in a KEGG functional element do not share similar functions. For instance, the G_i/o_ element contains 22 guanine-nucleotide-binding proteins, some of which belong to the G-alpha family (*GNAI1-3*, *GNAO1*), and others to the WD-repeat-G-beta family (*GNB1-5)*. Some function mainly as inhibitors (*GNAI*) while others are mainly engaged in activation (*GNAO*). Also, some elements contain a protein subunit that appears in every receptor, such as the *NR1* subunit in the NMDA receptor, and these should not be considered as CDVs.

To reduce detection of unrelated proteins as CDVs, we excluded protein pairs with amino-acid sequence similarity lower than 30%, since they are unlikely to share a similar function [36,37,75–77].

### Pairwise similarity between amino-acid sequences

Protein sequences were aligned using the Needleman-Wunsch global algorithm. Following the definition used for global alignment in BLAST 2 Sequences [78] we used blosum62 as the scoring matrix, and set the gap costs to 11 for gap existence and 1 for each extension. The sequence similarity was defined as the fraction of identical residues out of the number of residues in the longer sequence.

### Genome-wide analysis of paralogs

We analyzed all pairs of human genes pairs defined in Biomart on January 15th 2015 as paralogs and having entrez ids, yielding a total of 74984 pairs. We grouped these pairs by their sequence overlap as computed by Biomart, and computed the distribution of anti-correlation scores for each of the groups. We also computed the distribution of correlation *p*-values for a baseline set of 100K pairs, drawn uniformly at random from all gene measurements in the data that have an entrez id. Fig 1C and 1D depicts the distribution of pairs as a function of their correlation p-values for microarrays data (Fig 1C) and Brainspan RNA-seq data (Fig 1D).

### Quantify dissimilarity between expression profiles

To find genes that show opposite trend in their expression profile, we measured the Spearman correlation between the expression profiles of each pair of genes in every brain region. Analysis was limited to differentially expressed genes, by excluding genes whose expression range (maximum–minimum) was lower than 1.5 in log_2_ scale. We verified visually that this threshold excluded genes whose expression did not change considerably over time.

### Age-dependent correlation

The measure of correlation between two signals *x*_*t*_, *y*_*t*_, formally $\left(x_{t}-\bar{x})(y_{t}-\bar{y}\right)$, captures how the two signals jointly fluctuate around their corresponding means $\bar{x}=\frac{1}{n(x)}\sum \;x_{t}$ and $\bar{y}=\frac{1}{n(y)}\sum \;y_{t}$. However, when the signals are non-stationary, the mean of samples taken from a local period is itself changing within time. A natural idea is therefore to ‘detrend’ the data: learn models of the trend $\bar{x}(t),\bar{y}(t)$ and use them to compute the fluctuations around the trend $Correlation\_arround\_the\_trend=\left(x_{t}-\bar{x}(t)\right)\left(y_{t}-\bar{y}(t)\right)$. Importantly, this detrended correlation measure is very different from the correlation of the trends $Trend\_Correlation=(\bar{x}(t)-\bar{x})(\bar{y}(t)-\bar{y})$. This is illustrated in S5 Fig, where the trend correlation is negative–illustrated by the two solid lines, while the correlation around the trend is positive–illustrated by the arrows capturing the fluctuations around the lines. While the derivation above used Pearson (linear) correlations between the signals, any measure of correlation (like Spearman) can be applied to the trended data. In the context of the current paper the correlation of the trend capture the changes in expression through life at the population, and the correlations around the trend capture subject-to-subject fluctuations.

When the trend is modeled as a linear function, as in S5 Fig, the resulting correlation around the trend is known as *partial correlations*. Other, non-linear models have also been proposed. For instance, Podobnik and Stanley [41] have analyzed a piece-wise linear model, where time is divided to non-overlapping boxes, and each period is used to model the local trend. The current paper takes a more general approach to modelling the trend, using smoothing splines.

Specifically, to correct for age-dependent trends, we first learned a model of the expression profiles, by fitting a cubic smoothing spline to the expression profile of each gene separately as in [79]. The number of knots was set to the minimal number for which the spline satisfied the condition $\frac{1}{n}\sum_{i=1}^{n}{\left(y_{i}-f(x_{i})\right)}^{2}<{\hat{\sigma }}^{2}$, where ${\hat{\sigma }}^{2}$ is the estimated variance of the residuals computed by taking the mean of the variances for a sliding window of 10 data points. In a small minority of cases this condition could not be satisfied. In those cases, we relaxed the bound by using $2\hat{\sigma }$. Fig 3A illustrates the spline fitting for two Serotonin genes.

We used a leave-one-sample-out procedure to estimate the goodness of fit. Specifically, for each data point *i*, we computed the squared error for the predicted value ${\hat{y}}_{i}$ computed by fitting the model with all points except point *i*. The goodness of fit was then computed as $R^{2}=1-\frac{\sum_{i=1}^{n}{\left(y_{i}-{\hat{y}}_{i}\right)}^{2}}{\sum_{i=1}^{n}{\left(y_{i}-\bar{y}\right)}^{2}}$, where $\bar{y}=\frac{1}{n}\sum_{i=1}^{n}y_{i}.\;R^{2}$ measures by how much we reduce the variance compared to a baseline model of ${\hat{y}}_{i}=\bar{y}$. It is bounded by 1 and is usually positive, since the spline can assume the form ${\hat{y}}_{i}=\bar{y}$ as a special case. A score around zero usually means that the data show no trend and some fits have a negative score due to overfitting, since we are evaluating the fit using held out data. S2 Fig shows the distribution of *R* ^2^ values attained by the model compared to the distribution over the same data after shuffled in time. The spline models were on average superior to regularized polynomial regression using polynomials of degrees 1 to 3.

## Supporting Information

S1 Fig CDVs in glutamate and GABA receptors. **(A)** The gene expression profiles of *GRIN2B* and *GRIN2C* as measured by [32] in the human cerebellum. The expression level of *GRIN2B* (*NR2B*) declines during prenatal and early childhood, as opposed to the expression level of *GRIN2C* (*NR2A*) which rises during the same period. **(B)** The gene expression profiles of *GABRA3* and *GABRA6* in the human cerebellum, showing a similar switch. **(C)** & **(D)** RNA-seq data [34].
(DOCX)

S2 Fig Quality of fitting a cubic-spline as a population trend model. Quality is evaluated using test-set R^2^ (See Methods), which corresponds to the fraction of explained variance captured by the model. Negative values reflect overfitting, a value of zero means that no significant trend is captured by the model. Blue histogram: the distribution of the test-R2 over all genes in the examined dataset. Green: test-set R2 computed over randomly permuted data points for each gene.
(DOCX)

S3 Fig Dissimilarity significance based on RNA-seq of the top 20 genes in 16 brain regions provided by Brainspan. Colors correspond to negative log_*10*_ (*p*-values) of Pearson correlation. Gray pixels denote regions in which the range of expression levels (maximum-minimum) was below 1.5 for at least one gene, or an insignificant *q-value*. The bottom row shows dissimilarity for an interesting pair of serotonin receptors. Brain regions are grouped and sorted as follows: prefrontal: DFC, OFC, VFC, MFC; frontal-parietal: M1C, S1C, IPC; temporal-occipital: ITC, STC, A1C, V1C; subcortical: AMY, CBC, HIP, MD, STR. Region codes are listed in the Methods section.
(DOCX)

S4 Fig Agreement of correlation values between RNA seq (brainspan) and microarrays (Kang 2011) data. Each dot corresponds to a pair of genes. **Left:** trend correlations. **Right:** age-corrected correlation.
(DOCX)

S5 Fig A schematic illustrating the idea of age-corrected correlations. The expression measurements of two genes are shown (red and blue circles), together with the underlying trend (red and blue solid lines). The expression levels of the two genes are anti correlated, red being expressed more than blue in childhood, but less when older. However, when considering the difference between the measured samples and the trend, these residuals (red and blue vertical arrows) are positively correlated.
(DOCX)

S6 Fig Fraction of pairs achieving a significant *p*-value of the age-corrected correlation as a function of the significance thresholds. Gene pairs from the set of brain-related pathways are ~10 times more likely to achieve a significant correlation as compared to a baseline of house-keeping genes, for thresholds of log10(p)>7. Correlations were computed based on a spline model of Brainspan 2014 data (RNA-seq).
(DOCX)

S1 Text Supplemental analysis. Estimating the potential contribution of sampling fluctuations to age-corrected correlations.
(DOCX)
