# Supplementary material for: Gene Expression Switching of Receptor Subunits in Human Brain Development
Source: PLoS Comput Biol. 2015 Dec 4;11(12):e1004559. doi: 10.1371/journal.pcbi.1004559 (PMC4670163; doi:10.1371/journal.pcbi.1004559)
Supplement: S1 Fig — (A) The gene expression profiles of GRIN2B and GRIN2C as measured by [32] in the human cerebellum. The expression level of GRIN2B (NR2B) declines during prenatal and early childhood, as opposed to the expression level of GRIN2C (NR2A) which rises during the same period. (B) The gene expression profiles of GABRA3 and GABRA6 in the human cerebellum, showing a similar switch. (C) & (D) RNA-seq data [34]. (DOCX) [file pcbi.1004559.s001.docx]

| 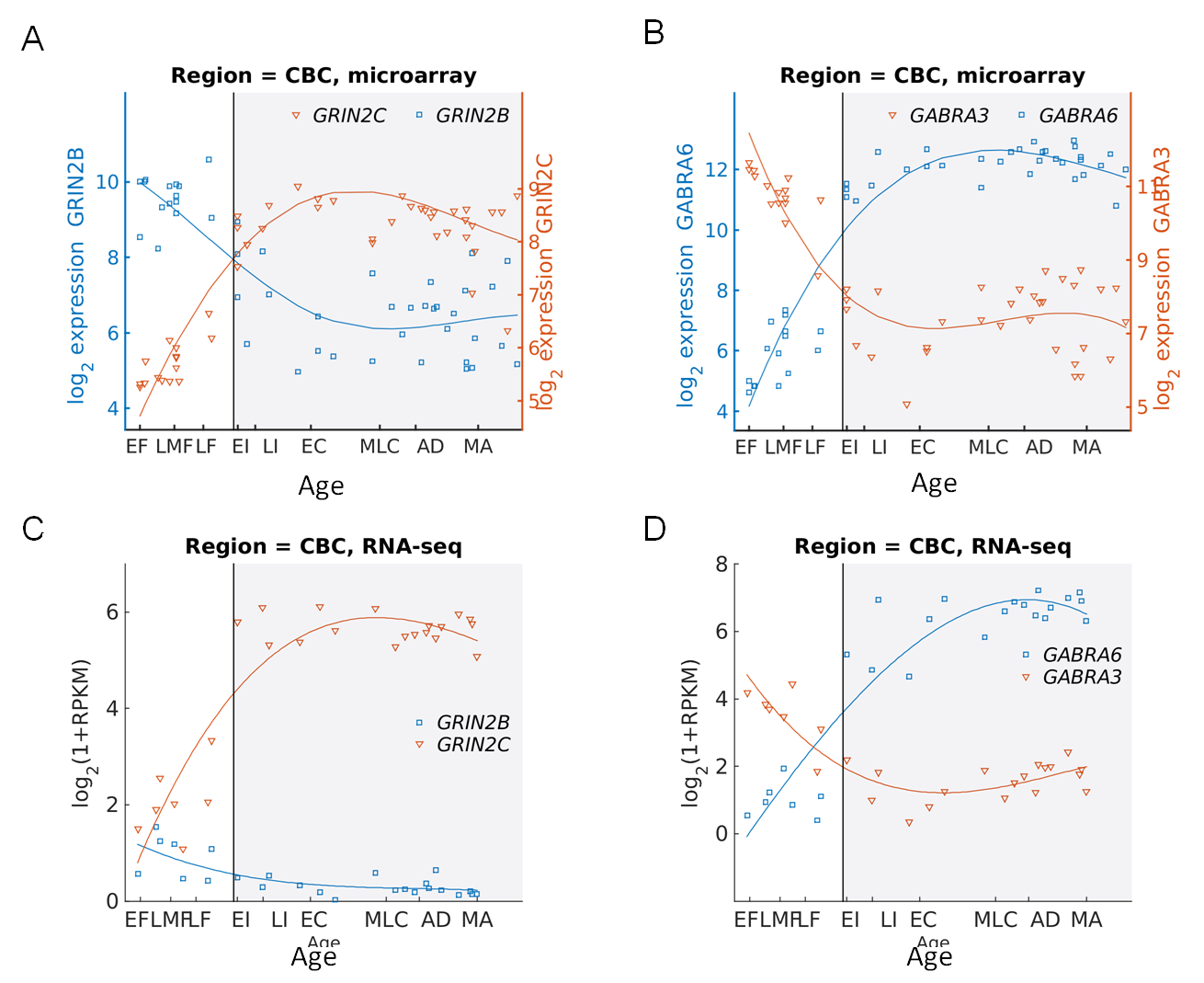 |
| --- |
| **Supporting Figure S1:** CDVs in glutamate and GABA receptors. **(A)** The gene expression profiles of *GRIN2B* and *GRIN2C* as measured by [32] in the human cerebellum. The expression level of *GRIN2B* (*NR2B*) declines during prenatal and early childhood, as opposed to the expression level of *GRIN2C* (*NR2A*) which rises during the same period. **(B)** The gene expression profiles of *GABRA3* and *GABRA6* in the human cerebellum, showing a similar switch. **(C)** & **(D)** RNA-seq data [74]. |
