# Supplementary material for: Gene Expression Switching of Receptor Subunits in Human Brain Development
Source: PLoS Comput Biol. 2015 Dec 4;11(12):e1004559. doi: 10.1371/journal.pcbi.1004559 (PMC4670163; doi:10.1371/journal.pcbi.1004559)
Supplement: S2 Fig — Quality is evaluated using test-set R2 (See Methods), which corresponds to the fraction of explained variance captured by the model. Negative values reflect overfitting, a value of zero means that no significant trend is captured by the model. Blue histogram: the distribution of the test-R2 over all genes in the examined dataset. Green: test-set R2 computed over randomly permuted data points for each gene. (DOCX) [file pcbi.1004559.s002.docx]

| 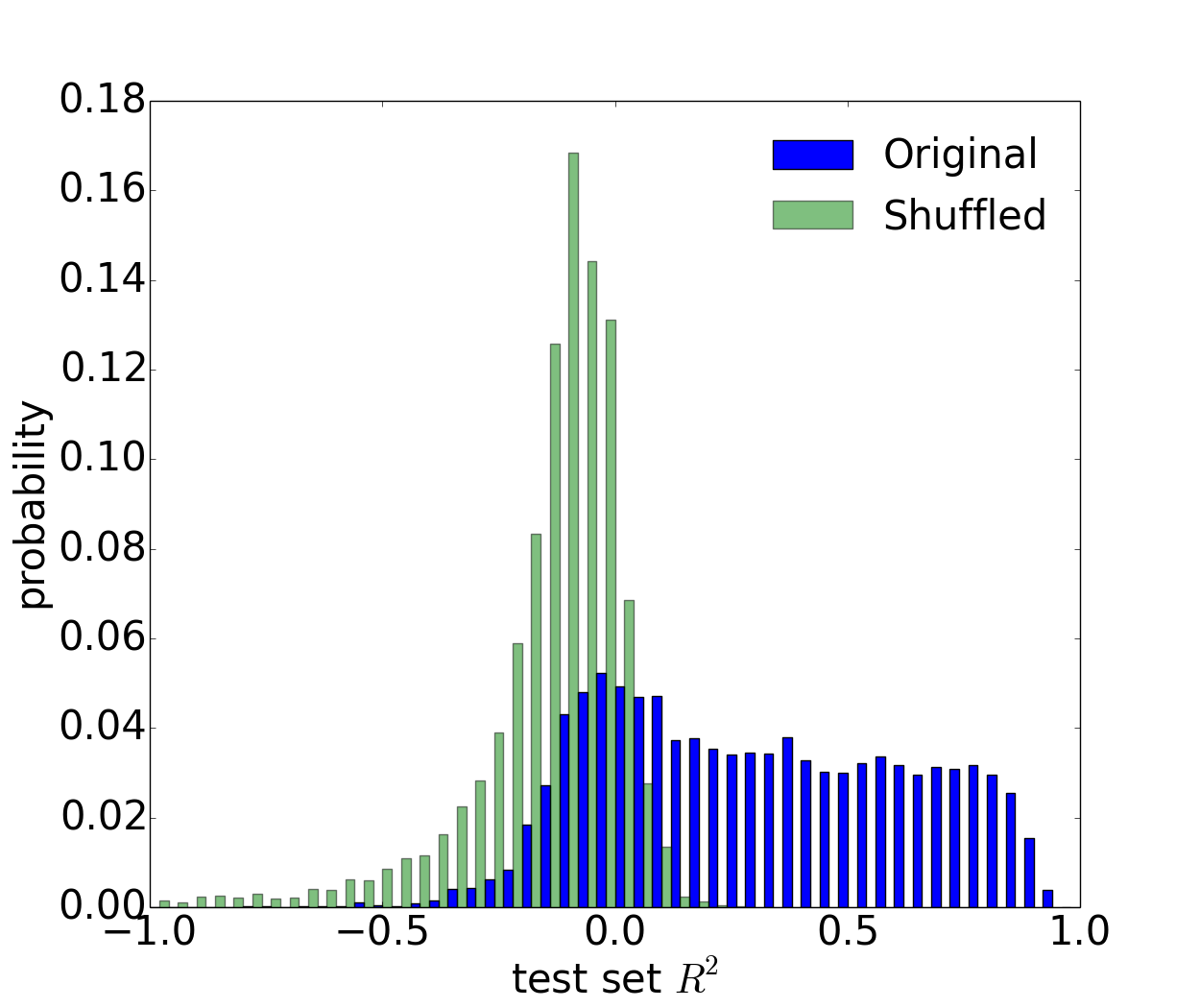 |
| --- |
| **Supporting Figure S2:** Quality of fitting a cubic-spline as a population trend model. Quality is evaluated using test-set R^2^ (See Methods), which corresponds to the fraction of explained variance captured by the model. Negative values reflect overfitting, a value of zero means that no significant trend is captured by the model. Blue histogram: the distribution of the test-R2 over all genes in the examined dataset. Green: test-set R2 computed over randomly permuted data points for each gene. |
