# Supplementary material for: Gene Expression Switching of Receptor Subunits in Human Brain Development
Source: PLoS Comput Biol. 2015 Dec 4;11(12):e1004559. doi: 10.1371/journal.pcbi.1004559 (PMC4670163; doi:10.1371/journal.pcbi.1004559)
Supplement: S3 Fig — Colors correspond to negative log10 (p-values) of Pearson correlation. Gray pixels denote regions in which the range of expression levels (maximum-minimum) was below 1.5 for at least one gene, or an insignificant q-value. The bottom row shows dissimilarity for an interesting pair of serotonin receptors. Brain regions are grouped and sorted as follows: prefrontal: DFC, OFC, VFC, MFC; frontal-parietal: M1C, S1C, IPC; temporal-occipital: ITC, STC, A1C, V1C; subcortical: AMY, CBC, HIP, MD, STR. Region codes are listed in the Methods section. (DOCX) [file pcbi.1004559.s003.docx]

| 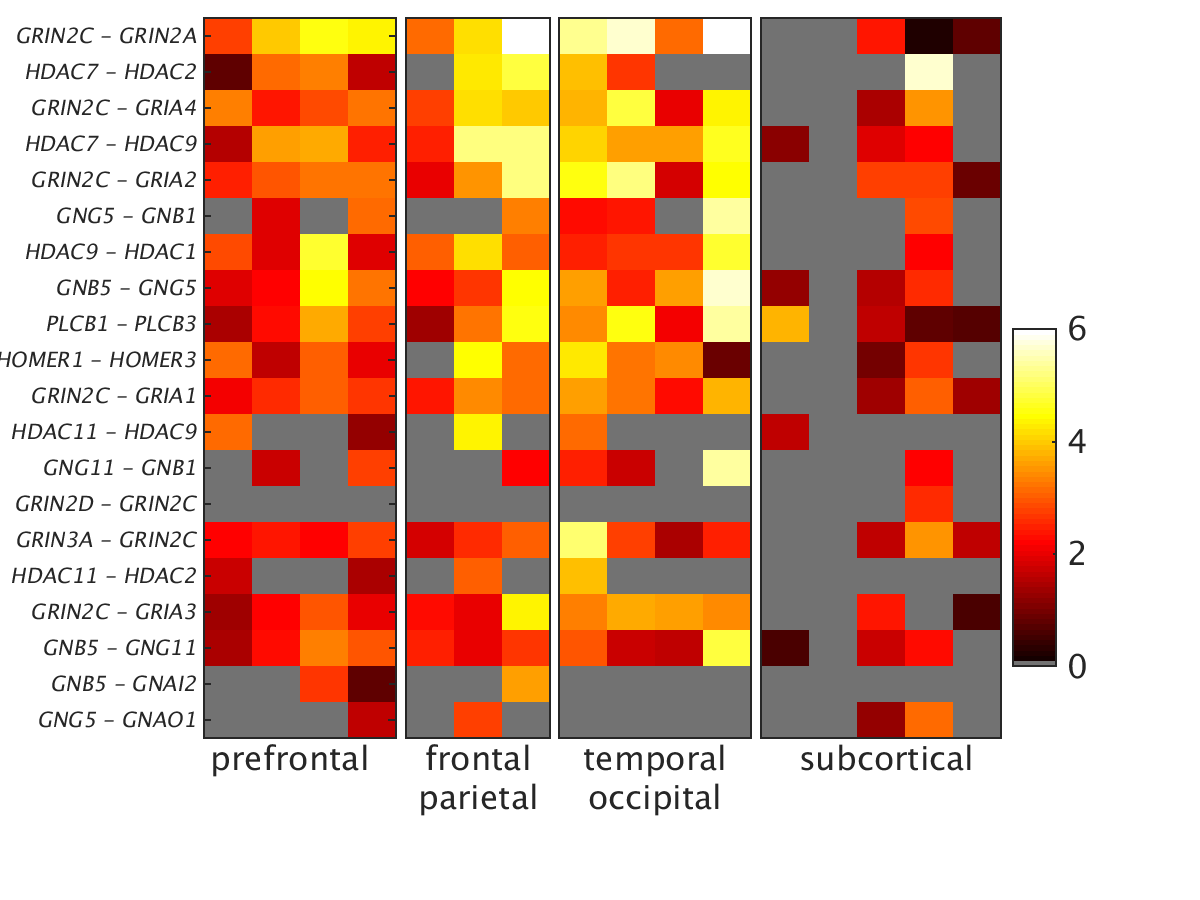 |
| --- |
| **Supporting Figure S3**. Dissimilarity significance based on RNA-seq of the top 20 genes in 16 brain regions provided by Brainspan. Colors correspond to negative log*_10_* (*p*‑values) of Pearson correlation. Gray pixels denote regions in which the range of expression levels (maximum-minimum) was below 1.5 for at least one gene, or an insignificant *q-value.* The bottom row shows dissimilarity for an interesting pair of serotonin receptors. Brain regions are grouped and sorted as follows: prefrontal: DFC, OFC, VFC, MFC; frontal-parietal: M1C, S1C, IPC; temporal-occipital: ITC, STC, A1C, V1C; subcortical: AMY, CBC, HIP, MD, STR. Region codes are listed in the Methods section. |
