# Supplementary material for: Gene Expression Switching of Receptor Subunits in Human Brain Development
Source: PLoS Comput Biol. 2015 Dec 4;11(12):e1004559. doi: 10.1371/journal.pcbi.1004559 (PMC4670163; doi:10.1371/journal.pcbi.1004559)
Supplement: S4 Fig — Each dot corresponds to a pair of genes. Left: trend correlations. Right: age-corrected correlation. (DOCX) [file pcbi.1004559.s004.docx]

| 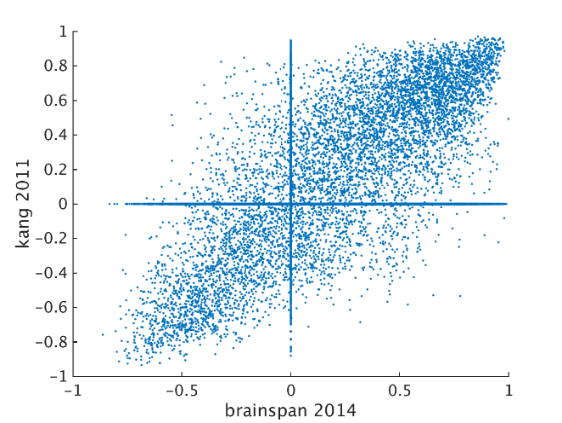 | 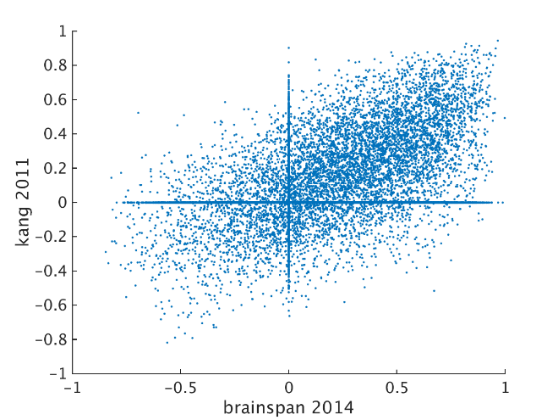 |
| --- | --- |
| **Supporting Figure S4:** Agreement of correlation values between RNA seq (brainspan) and microarrays (Kang 2011) data. Each dot corresponds to a pair of genes. **Left:** trend correlations. **Right:** age-corrected correlation. | |
