# Supplementary material for: Gene Expression Switching of Receptor Subunits in Human Brain Development
Source: PLoS Comput Biol. 2015 Dec 4;11(12):e1004559. doi: 10.1371/journal.pcbi.1004559 (PMC4670163; doi:10.1371/journal.pcbi.1004559)
Supplement: S5 Fig — The expression measurements of two genes are shown (red and blue circles), together with the underlying trend (red and blue solid lines). The expression levels of the two genes are anti correlated, red being expressed more than blue in childhood, but less when older. However, when considering the difference between the measured samples and the trend, these residuals (red and blue vertical arrows) are positively correlated. (DOCX) [file pcbi.1004559.s005.docx]

| 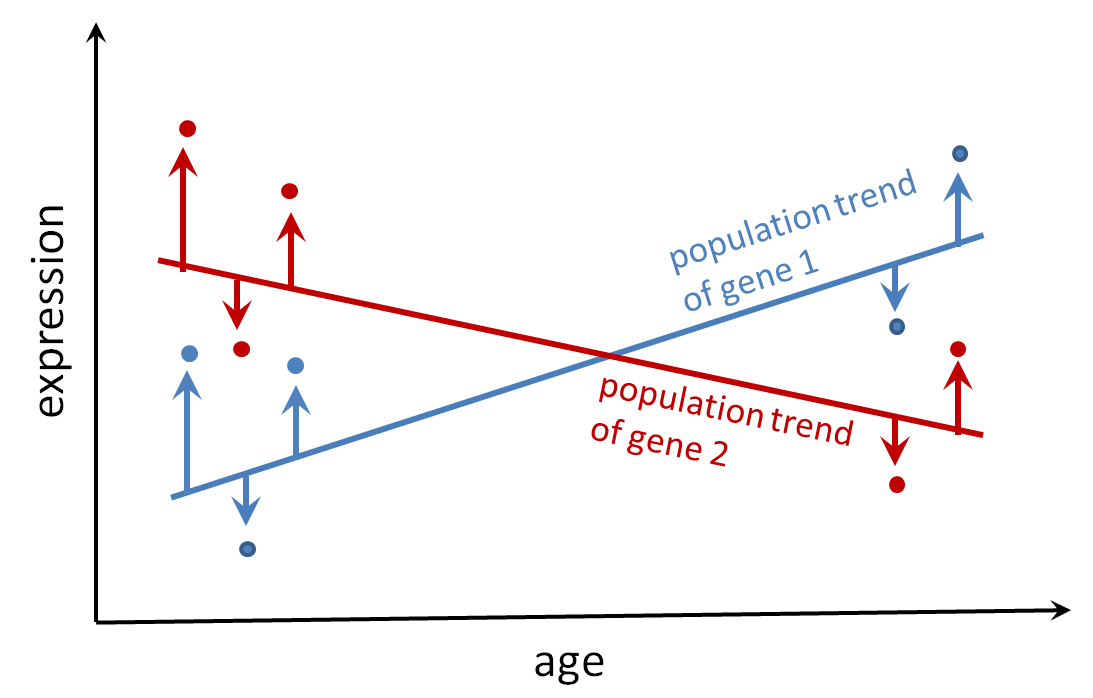 |
| --- |
| **Supporting Figure S5:** A schematic illustrating the idea of age-corrected correlations. The expression measurements of two genes are shown (red and blue circles), together with the underlying trend (red and blue solid lines). The expression levels of the two genes are anti correlated, red being expressed more than blue in childhood, but less when older. However, when considering the difference between the measured samples and the trend, these residuals (red and blue vertical arrows) are positively correlated. |
