# Supplementary material for: Gene Expression Switching of Receptor Subunits in Human Brain Development
Source: PLoS Comput Biol. 2015 Dec 4;11(12):e1004559. doi: 10.1371/journal.pcbi.1004559 (PMC4670163; doi:10.1371/journal.pcbi.1004559)
Supplement: S6 Fig — Gene pairs from the set of brain-related pathways are ~10 times more likely to achieve a significant correlation as compared to a baseline of house-keeping genes, for thresholds of log10(p)>7. Correlations were computed based on a spline model of Brainspan 2014 data (RNA-seq). (DOCX) [file pcbi.1004559.s006.docx]

| 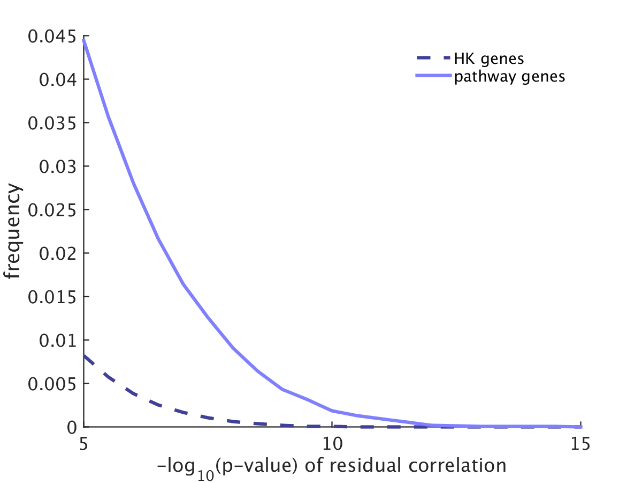 |
| --- |
| **Supporting Figure S6**. Fraction of pairs achieving a significant *p*-value of the age-corrected correlation as a function of the significance thresholds. Gene pairs from the set of brain-related pathways are ~10 times more likely to achieve a significant correlation as compared to a baseline of house-keeping genes, for thresholds of log10(p)>7. Correlations were computed based on a spline model of Brainspan 2014 data (RNA-seq). |
