# Supplementary material for: Gene Expression Switching of Receptor Subunits in Human Brain Development
Source: PLoS Comput Biol. 2015 Dec 4;11(12):e1004559. doi: 10.1371/journal.pcbi.1004559 (PMC4670163; doi:10.1371/journal.pcbi.1004559)
Supplement: S1 Text — Estimating the potential contribution of sampling fluctuations to age-corrected correlations. (DOCX) [file pcbi.1004559.s007.docx]

**Supplemental analysis**

We wanted to test what part of the age-corrected correlations could results from fluctuations in sampling. These could be for instance due to variable ratio of glia to neurons across subjects.

We estimated the potential effect of such subject-to-subject sampling variability on age-corrected correlations using a simple model. In our model, pairs of uncorrelated expression profiles were created, with the same number of samples as in our data (57 samples per profile). Each sample (corresponding to a subject) was corrupted by a multiplicative Gaussian noise$\xi\sim\mathcal{N}(1,\sigma^{2})$, affecting both genes. We then computed the fraction of pairs that achieve strong correlations as a function of $\sigma$. To reach the same fraction of pairs with correlation with p-value stronger than 10^-5^, $\sigma$ had to be larger than 40%. However, with such strong fluctuations, 97% of the pairs were positively correlated, while the real data has an order of magnitude more negatively correlated pairs. This suggests that the positive residual correlations observed in our data are not due to a genome-wide effect but are more specific to the set of gene studied.
